# P089: Surveillance of bacterial resistance to disinfectants

**DOI:** 10.1186/2047-2994-2-S1-P89

**Published:** 2013-06-20

**Authors:** N Saperkin, O Kovalishena, A Blagonravova

**Affiliations:** 1 Epidemiology, Nizhniy Novgorod State Medical Academy, Nizhniy Novgorod, Russian Federation

## Introduction

The causative agents of healthcare-associated infections are known to be resistant both to antibiotics, disinfectants, and antiseptics. Our sampling researches allowed to implement a monitoring in hospitals.

## Objectives

The aim was to study susceptibility of the microflora in 2009-2011 in order to correct disinfection in good time.

## Methods

Our own microbiologic method (patent RF №2378363, 2008) was used for evaluating the bacterial susceptibility to disinfectants. Comparative statistical analysis was made by EpiInfo v.7.

## Results

On average 40 hospitals per year take part in the monitoring. The cultures are searched to more than 50 trademarks of biocides. In 2011 we tested 445 strains. Most of bacteria were susceptible to disinfectants of the different chemical classes in 2011: 82% were susceptible to biocides based on "quaternary ammonium compounds (QAC)+guanidine", 100% - to tertiary amines etc. Total incidence of resistant strains was 3.4±3.0 per 100 tests. It was to 4 chemical classes of disinfectants. The biggest share of resistant cultures was to the oxygen-contained biocides - 6.3% of 29 strains tested to this class. On the contrary, the minimal level was to disinfectants containing QAC+amines (1.7%, n=58).

The resistant strains Acinetobacter sp were predominant (13% of all cultures of the species). Moreover, the percentage of resistant Pseudomonas aeruginosa was 5.7% of all cultures of the species. We have also revealed resistance to disinfectants in Escherichia coli, Proteus vulgaris, Proteus mirabilis.

In dynamics we found out increase in the incomplete susceptibility of bacteria to different QACs (p=0.3). The incidence of such susceptibility to chlorine compounds was significantly lower vs in 2009 and 2010 (p=0.038). In 2011 there were 2.9 times more resistant bacteria to oxygen-contained disinfectants (p=0.03). During all years no resistant strains to chlorine compounds were revealed. The amount of Pseudomonas aeruginosa decreased 2 times (p=0.06 vs 2009) but it was due to the reduced number of the strains we have tested.

## Conclusion

There were resistant strains to QACs and oxygen-contained disinfectants which are widely used in the hospitals of the Nizhniy Novgorod region. The monitoring of resistance to biocides provides an operative evaluation of the microbial susceptibility in healthcare settings.

## Disclosure of interest

None declared

